# Giant lateral left ventricular wall aneurysm sparing the submitral apparatus

**DOI:** 10.1186/1749-8090-8-201

**Published:** 2013-10-30

**Authors:** Andreas Habertheuer, Martin Andreas, Dominik Wiedemann, Claus Rath, Alfred Kocher

**Affiliations:** 1 Division of Cardiac Surgery, Vienna General Hospital, Medical University of Vienna, Waehringer Guertel 18-20, A-1090 Vienna, Austria

**Keywords:** Giant left ventricular aneurysm, Ischemic stroke, Motor aphasia with unilateral sensorimotor deficits, Surgical ventricular restoration, Dor procedure

## Abstract

Left ventricular aneurysms are a frequent and serious complication following acute transmural myocardial infarction and are most commonly located at the ventricular apex. The majority of these patients presents with severe mitral insufficiency, congestive heart failure, systemic embolism and sudden cardiac death. Giant aneurysms occurring in a submitral position between anterior and posterior papillary muscles on the lateral ventricular wall constitute a minor entity and those leaving the mitral apparatus intact are extremely rare.

Herein, we report the case of a 57 y/o Caucasian male patient with a past medical history of coronary artery disease and myocardial infarction with a giant left ventricular aneurysm measuring 15x10x8 cm in diameter. Despite the size of the aneurysm and its close topographical relation to the posterior mitral annulus the mitral apparatus was intact with a competent valve and normal left atrial size. He underwent successful surgical ventricular restoration.

## Background

True left ventricular aneurysms are widely recognized as a common and serious complication following total occlusion of coronary arteries [1-4] with a prevalence of approximately 12% of acute transmural myocardial infarctions according to a series of 508 autopsies performed by Abrams et al. in the Micheal Reese Hospital and Medical Center, Chicago, Illinois [5]. According to the available scientific literature [5] the apex and the posterior wall are the most common sites of aneurysm formation (31.7% and 23.8%, respectively). The anterior wall and the intraventricular septum are less frequently involved (9.5% and 7.9%, respectively). As few as 1% of ventricular aneurysms affect the lateral wall of the ventricle and are usually not extensive.

Ventricular aneurysm is a serious disorder with a varied clinical presentation depending on size, location and valvular involvement, making this etiology difficult to diagnose. Large aneurysms profoundly change cardiac geometry, impair systolic and diastolic function, result in dyskinesia or do have arrhythmic potential. Distortion of the mitral and aortic annuli produces incompetence of these valves while compression of a coronary artery may cause coronary insufficiency or occlusion [6]. An altered endocardial morphology my further predispose to systemic emboli secondary to mural thrombi that form in the aneurysmal sac and fatal ventricular rupture.

## Case presentation

A 57 y/o Caucasian male patient was referred to our hospital with pronounced motor aphasia and unilateral right sensorimotor performance deficits after having been found unconscious at home. In the ER the patient was awake and oriented to person, place and time. Due to the severe dynamic aphasia and agitation it was not possible to obtain a sufficient patient history. According to the patient’s relatives the onset of neurological symptoms was the evening before, however less pronounced and progressed to an unconscious state the next morning. Initially the patient and his family denied any history of chest pain, orthopnea, and lower extremities edema, however eventually the patient recalled an episode of chest pain and lightheadedness as angina pectoris equivalent two years before. Coronary angiography revealed a three vessel disease with a total occlusion of the first marginal branch of circumflex artery which apparently lead to transmural infarction and subsequent aneurysm formation. His past medical history was otherwise remarkable for essential hypertension and intermittent atrial fibrillation. On physical exam his pupils were equal round and reactive to light and accommodation. He manifested a unilateral faciobrachial weakness as well as a unilateral faciobrachial sensory loss. His cardiac risk factors were significant for tabacco abuse (50 pack years), essential hypertension, unhealthy diet and physical inactivity.

**Figure 1:**
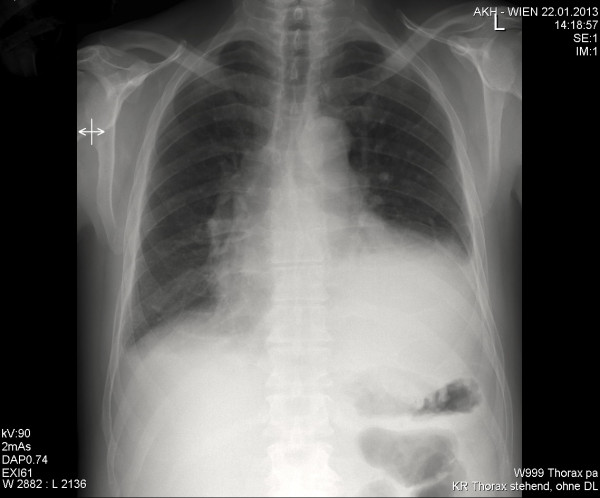
**Preoperative chest X-ray, showing cardiomegaly suspicious of true left ventricular aneurysm complicating myocardial infarction.** Frontal view. Abnormal evagination of left cardiac border is typical for an aneurysm involving the anterolateral and/or apical segment of the left ventricle. The aneurysm bulges out and stretches to the thoracic wall. There is associated pulmonary congestion due to heart failure.

The electrocardiogram showed atrial fibrillation at 120 beats/min and pathologic Q-waves in leads I and aVL consistent with a non-recent lateral myocardial infarction. The tachycardic supraventricular arrhythmia was successfully converted with 300 mg of amiodarone. Cranial computed tomography was immediately initiated and revealed a large left frontotemporal and right parietal hypoattenuation consistent with cerebral infarction. The patient was started on high molecular weight heparin. Chest X-rays showed a massive cardiomegaly with a bulging protrusion along the left ventricular shadow (Figure 1). Comparison to prior X-ray studies raised the suspicion of a giant ventricular aneurysm. There was associated pulmonary venous congestion due to heart failure.

**Figure 2:**
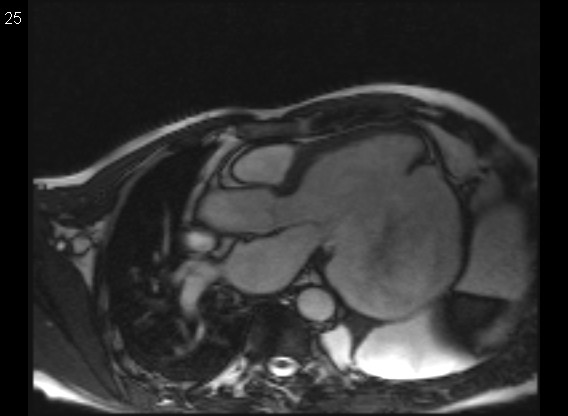
**Preoperative magnetic resonance imaging.** Left ventricular outflow tract. Giant left ventricular aneurysm of the lateral wall, strictly confined to the submitral region between anterolateral and posteromedial papillary muscle. Narrow aneurysma neck stretching in length from the apex up to 1 cm below the mitral valve annulus with the aneurysmal sac bulging to the thoracic wall. Signal alteration as sign of blood stasis in left ventricle. Spontaneous contrast on echocardiography. Of note: thin aneurysmal sac.

The preoperative echocardiographic evaluation and magnetic resonance imaging confirmed the diagnosis of a 15×10×8cm left ventricular aneurysm along the left ventricular wall (Figure 2 and Additional file 1: Video 1). Spontaneous echo contrast was clearly visible as a dynamic sign of blood stasis in the aneurysmal sac. Despite the huge size of the aneurysm the mitral valve was competent due to the fact that the neck of the aneurysm which extended from the base of the heart to the apex and was located exactly in-between the anterolateral und posteromedial papillary muscles. This way the submitral apparatus was spared from any tethering and necrosis. Again the neck was only 2 cm wide but 8 cm long, stretching in length from the apex up to 1 cm below the mitral valve annulus with the aneurysmal sac bulging to the thoracic wall (Figure 2). The left ventricular (LV) ejection fraction was estimated at 10-15% with an akinetic lateral wall and a grade one LV diastolic dysfunction. No intracardiac thrombus was appreciated, however a sponatanous contrast was visible in the aneurysmal sac. All four valves were competent and the estimated pulmonary artery systolic pressure was 24 mm Hg.

Cardiac catheterization showed 3-vessel disease with 60% stenosis of the proximal right coronary artery (RCA) as well as a 60% stenosis of the proximal posterior descending artery, a 30% stenosis of the left anterior descending artery (LAD), a 75% stenosis of the first diagonal branch and a proximal total occlusion of the first obtuse marginal branch the circumflex artery (OM).

**Figure 3:**
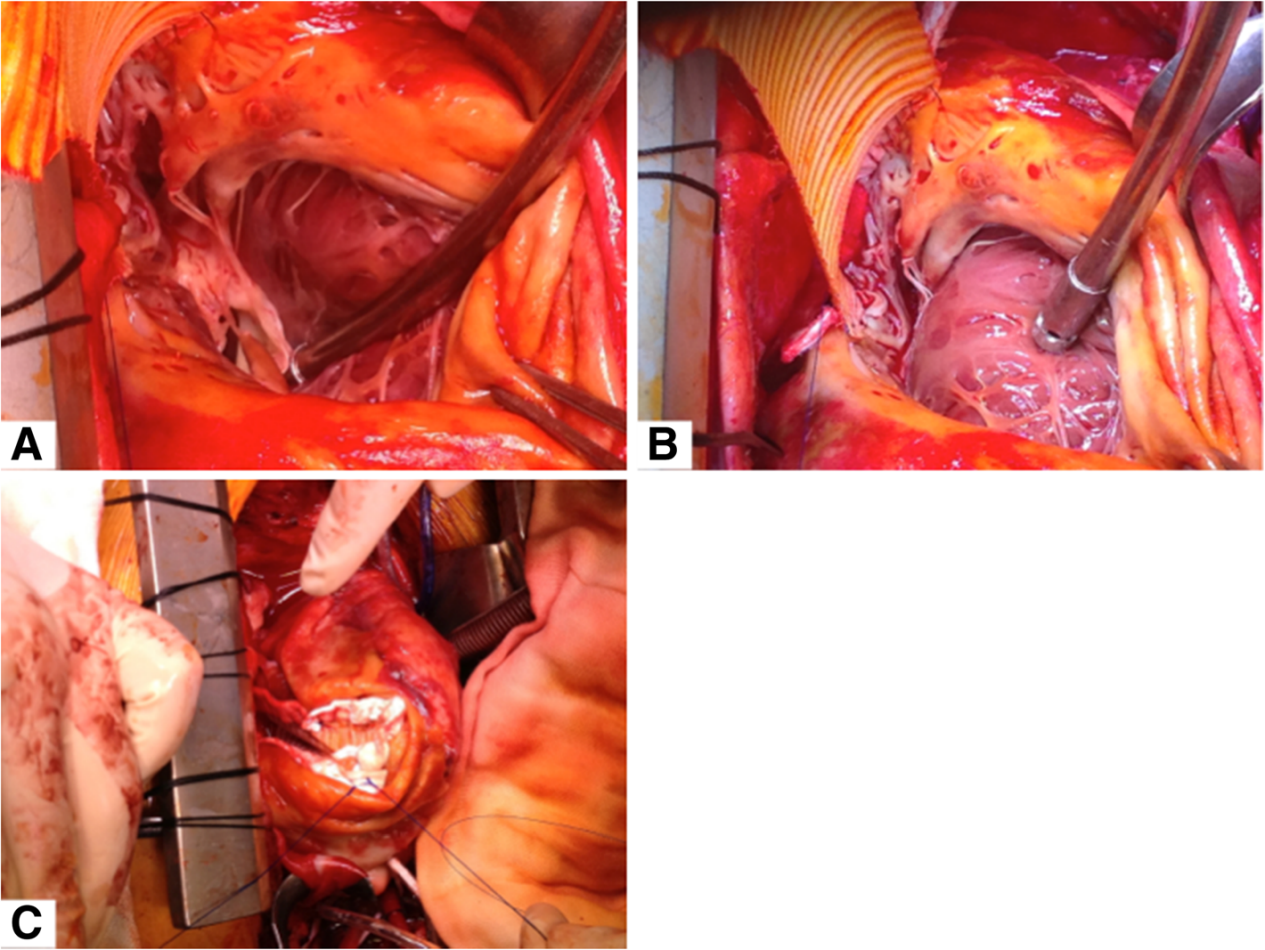
**Intraoperative situs upon median sternotomy. A** Excision of ventricular aneurysm prior to reconstruction. Ventricular endocard visible. **B** Surgical lateral ventricular restoration with a Dacron patch tailored from a 34 mm tube graft in order to mimick the geometry of the heart. **C** Situs upon ventricular restoration using a Dacron patch.

The patient was in NYHA class III. He was diagnosed with a giant ventricular aneurysm 2 years after his myocardial infarction and underwent surgical lateral ventricular restoration 2 months after the initial neurologic presentation. The patient was put on pump via a standard bicaval cannulation and aortic cannulation. A left ventricular vent was installed via the right superior pulmonary vein. There were extensive adhesions between the heart and entire pericardium that could only be detached after aortic cross-clamping. Care was taken to keep the 2 mm thin aneurysm wall intact for later hemostasis. After liberating the heart from the pericardial sac the aneurysm was incised and the situs inspected. The entry of the aneurysm had a neck between the papillary muscles and extended from the apex to within 1 cm of the posterior mitral valve leaflets (Figure 3A). Ventricular restoration was done using the Dor procedure with a Dacron patch tailored from a 34 mm tube graft in order to mimick the geometry of the heart (Figure 3B). The thin wall of the giant aneurysm was partially resected and the limbus was sown together buttressed with a felt strip with 3/0 prolene for hemostatic purposes (Figure 3C). Thereafter coronary revascularization was performed with a saphenous vein graft to the first diagonal branch and the right coronary artery, respectively. Myocardial protection was achieved with antegrade aortic and vein graft blood cardioplegia, as well as retrograde blood cardioplegia via the coronary sinus.

The patient was off all inotropes and pressors on postoperative day two and transferred to an outside neurology department on post-operative day ten. He was eventually discharged home in good clinical condition and with almost normal ejection fraction. Of note, the neurological situation improved dramatically with only minor sequelae. The patient was followed 9 months upon surgery, his neurological sequelae continued to improve with no residual cardiac impairment.

## Conclusion

The recognition of ventricular aneurysms is of great importance due to the numerous complications that can potentially occur. These complications consist primarily of intractable congestive heart failure, systemic emboli secondary to mural thrombi in the aneurysmal sac and ventricular rupture. In the case presented herein the neurological deficit was the first symptom of giant left ventricular aneurysm secondary to transmural myocardial infarction.

The concept of excluding the akinetic aneurysmal portion of the ventricle derives from the fact that when a portion of the ventricular wall is transformed into scar tissue and starts to dilate due to the high intraventricular pressure, the loss of function is higher then estimated by the loss of contractile tissue alone [7-9]. This is due to change in the ventricular geometry and loss of synchronization of muscle contraction that fundamentally alters the mechanics of the ventricular ejection phase [10]. It was noted that excluding that portion by resection could improve cardiac function by restoring ventricular geometry. Cooley described a resection and linear closure of aneurysms in 1958; Jatene described a technique of septal imbrication in 1985 and Cooley described a technique for septal exclusion in 1988. It was not, however, until Dor in 1985 that the concept of preservation of left ventricular geometry after such resections/exclusions became clear [10]. A timely diagnosis and early surgical treatment is vital in patient management to prevent fatal cardiac rupture and to prevent a decrease of cardiac function beneath a threshold where surgery would not be beneficial for patient outcome.

Perioperative ejection fraction is an important determinant for survival. Patients with a preoperative EF > 30% had a 77% 5-year survival while patients with an EF < 30% had a 64% survival chance [11]. In our patient the ejection fraction was restored to an almost normal value.

The overall 5 year survival rate following the Dor procedure is 69%, patients younger then 70 years of age had a 5-year survival of 70%, while older patients had only a 59% actuarial survival [12,13]. The Dor procedure has a perioperative mortality rate of 5.6% and requires a hospital stay of approximately 8 days which is only one day longer than for CABG [12]. Because the Dor procedure restores the left ventricle to its correct, elliptical shape it results in a mean ejection fraction increase of 12.5% [12]. This number continuous to improve over the patent’s lifetime and life expectancy is estimated to increase by 4–10 years [13].

In this case of giant left ventricular aneurysm following ischemic cardiomyopathy surgical ventricular restoration resulted in a normal shape of the ventricle and is supposed to result in a good long-term outcome.

## Consent

Written informed consent was obtained from the patient for publication of this case report and any accompanying images. A copy of the written consent is available for review by the Editor-in-Chief of this journal.

## Abbreviations

CABG: Coronary artery bypass graft; CT: Computed tomography; CXA: Circumflex artery; EF: Ejection fraction; ER: Emergency room; LCA: Left coronary artery; LMS: Left main stem; MRI: Magnetic resonance imaging; NYHA: New York heart association; OM: Obtuse marginal branch; PAP: Pulmonary arterial pressure; RCA: Right coronary artery; TOE: Transesophageal echocardiography; y/o: Years old.

## Competing interests

The authors declare that they have no competing interests.

## Authors’ contributions

AH assisted during surgery and prepared the manuscript. MA aided in literature search, participated in surgery. DW aided in literature search. CR aided in literature search. AK operating surgeon, drafted and reviewed the manuscript. All authors read and approved the final manuscript.

## Authors’ information

AH PhD student, Department of Cardiac Surgery, Vienna General Hospital, Medical University of Vienna. MA resident, Department of Cardiac Surgery, Vienna General Hospital, Medical University of Vienna. DW chief-resident, Department of Cardiac Surgery, Vienna General Hospital, Medical University of Vienna. CR PhD student, Department of Cardiac Surgery, Vienna General Hospital, Medical University of Vienna. AK professor of surgery, Department of Cardiac Surgery, Vienna General Hospital, Medical University of Vienna.

## Supplementary Material

Additional file 1: Video 1 Cardiac MRI. Magnetic resonance imaging shows a 15 x 10 x 8 cm left ventricular aneurysm along the left ventricular wall. Spontaneous echo contrast is clearly visible as a dynamic sign of blood stasis in the aneurysmal sac. Click here for file
